# Evaluation of tissue-engineered bone constructs using rabbit fetal osteoblasts on acellular bovine cancellous bone matrix

**DOI:** 10.14202/vetworld.2017.163-169

**Published:** 2017-02-08

**Authors:** Rashmi, Rekha Pathak, Amarpal, H. P. Aithal, P. Kinjavdekar, A. M. Pawde, A. K. Tiwari, P. Sangeetha, P. Tamilmahan, A. B. Manzoor

**Affiliations:** 1 Division of Veterinary Surgery, Indian Veterinary Research Institute, Izatnagar, Bareilly - 243 122, Uttar Pradesh, India; 2 Division of Standardization, Indian Veterinary Research Institute, Izatnagar, Bareilly - 243 122, Uttar Pradesh, India

**Keywords:** composite grafts, osteoblasts, tissue engineering

## Abstract

**Aim:**

The aim of this study was to generate composite bone graft and investigate the rabbit fetal osteoblasts adhesion, proliferation and penetration on acellular matrices of cancellous bone.

**Materials and Methods:**

Acellular cancellous bone was prepared and developed as in the previous study with little modification. These matrices were decellularized by rapid freeze and thaw cycle. To remove the cell debris, they were then treated with hydrogen peroxide (3%) and ethanol to remove antigenic cellular and nuclear materials from the scaffold. Primary osteoblast cells were harvested from 20 to 22 days old rabbit fetal long and calvarial bone. These cells were cultured and characterized using a specific marker. The third passaged fetal osteoblast cells were then seeded on the scaffold and incubated for 14 days. The growth pattern of the cells was observed. Scanning electron microscope and hematoxylin and eosin staining were used to investigate cells proliferation.

**Results:**

The cells were found to be growing well on the surface of the scaffold and were also present in good numbers with the matrix filopodial extensions upto inside of the core of the tissue.

**Conclusion:**

Thus, a viable composite scaffold of bone could be developed which has a great potential in the field of bone tissue engineering.

## Introduction

Many attempts have been made to develop a suitable bone graft substitute for repair of segmental bone defect. Segimental bone defects can be defined as injuries in which a section of bone is completely shattered or absent [1]. Restoration of skeletal function with autograft remains gold standard and most commonly used procedure. However, the disadvantages of autogenous bone grafts are limited availability, harvesting morbidity, and insufficient biomechanical properties [2]. The allogeneic bone graft may be associated with risk of pathogen transmission from donor to host, immunogenicity, and limited availability [3]. A resolution to the shortage of allograft tissues can be overcome by the use of xenografts [4]. Xenogeneic bone grafts consist of skeletal tissue that is harvested from one species and transferred to the recipient site of another species [5]. Since immune rejection is the greatest barrier to bone xenografts [6], the effective removal of antigenic epitopes associated with cell membranes and intracellular components of tissues and organs is necessary to minimize or avoid an adverse immunologic response by allogeneic and xenogeneic recipients of the extracellular matrix (ECM) scaffold material [7]. The physical method consisting of rapid cycle of freeze-thawing is found an efficient, reliable, quick, and easy method for the generation of acellular bone scaffold [8-10].

The advent of bone tissue engineering has brought new ideas and the development of innovative biomaterials for bone healing [11]. The principle of this method is to apply functionally active cells on supporting scaffolds under controlled stimulation with growth factors in order to produce biologic substitutes as functional tissue replacement [12]. It means a “composite graft” would contain osteogenic cells and osteoinductive growth factors along with an osteoconductive matrix. Osteoconductivty and osteoinductivity achieved when acellular bone matrix is used as scaffold but they fail to add osteogenic cells. Osteogenicity may be added to bioscaffolds by incorporating osteoblasts, mesenchymal stem cells, periosteal cells, or other osteogenic cell types [2,13-15]. Adult osteoblasts, even though widely characterized have typically limited expansion capabilities and can be harvested in small quantities posing limitations in cell availability [16]. One study has compared culture of fetal osteoblasts to primary adult osteoblasts and bone marrow osteoprogenitors and found that the fetal osteoblasts proliferate at higher rate, express higher level of alkaline phosphatase and are the true osteoblast progenitors [17]. Moreover, fetal cells are found to be having immunomodulatory activity [18].

Till date, the studies regarding seeding techniques of the xenogeneic acellular scaffolds with fetal osteoblasts, thereby making them ideal composite grafts, are actually lacking. Therefore, this study was designed to seed fetal osteoblasts on the acellular bone scaffold and to evaluate the uniformity of cell seeding and thus produce viable composite bone graft.

## Materials and Methods

### Ethical approval

The ethical permission was granted by our Institute Animal Ethical Committee, IVRI, Izatnagar.

### Equipment and instruments

The standard equipment and instruments available in Division of Surgery (DBT Lab), Division of Veterinary Public Health, Division of Animal Biotechnology, Division of Pathology, IVRI Bareilly and College of Veterinary Sciences, Govind Ballabh Pant University of Agriculture and Technology, Pantnagar were used for this study. Specifications of the instruments/equipment are given at the appropriate places in the text.

### Chemicals and reagents

The chemicals and cell culture media used for fetal osteoblast cultures were procured from the reputed agencies. The high glucose 1× Dulbecco’s modified Eagle’s medium (DMEM) media (Cat# 1233254) and streptomycin-penicillin (Cat#1670049) were procured from MP Biomedical Pvt. Ltd. Fetal bovine serum (FBS) (Cat# F2442), phosphate buffered saline (PBS) (Cat# PBS1), and 0.25% trypsin- ethylenediamine tetra acetic acid (EDTA) solution (Cat# T4049) were procured from Sigma-Aldrich, Pvt. Ltd. (St. Louis, USA).

### Xenogeneic acellular cancellous bone matrix

The acellular bone xenograft matrix was prepared from bovine femur by freeze and thaw cycles as described in previous studies [9,10]. Femur bone was procured from the slaughterhouse and cut into small pieces by osteotome. The bone pieces were thoroughly washed in tap water to remove the marrow contents and finally washed in 1× PBS with 0.1% EDTA at room temperature. These bone pieces were decellularized by 5 freeze and thaw cycles (one cycle consists of 1 min in liquid nitrogen and 5 min in hot water at 56°C). Furthermore, modification of this previous protocol was also done to reduce antigenicity. For that bone, pieces were kept into 3% H_2_O_2_ for 45 min [19] and enzymatic solutions to remove residual antigenic material. The scaffolds were sterilized by 70% ethanol and UV treatment. Sterility was checked by putting scaffolds in Dulbecco’s modified Eagle medium and incubated for 2 weeks to rule out any growth in media.

### Primary osteoblasts culture and seeding of cells on acellular matrix

**Figure-1:**
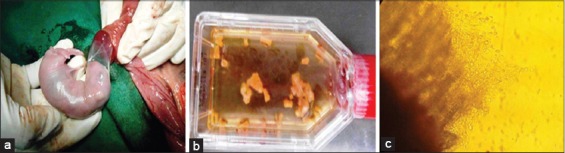
(a) 21 days old rabbit fetus, (b) Culture of digested fetal bone pieces, (c) Osteoblasts migrating from explants culture.

A healthy pregnant New Zealand White rabbit was utilized and after 21 days of gestation, fetuses were collected after cesarean section under general anesthesia induced with intramuscular injection of xylazine at 6 mg/kg followed 10 min later, by ketamine at 60 mg/kg [20]. Fetuses were collected in sterile 1% PBS (Figure-1a). Calvarial and long bone were harvested, cut into small pieces and washed with three times the usual concentration of antibiotics that is penicillin (100 U/ml) and streptomycin (100 mcg/ml) in 1% PBS. They were subjected to the enzymatic digestion in 0.25% trypsin at 37°C for 20 min [21]. The bone pieces were washed in a culture medium without serum after trypsinization procedure and then dipped in culture medium (DMEM) with 20% FBS, and they were laid equally in T-25 culture flask and incubated in a humidified atmosphere with 5% CO_2_ at 37°C for overnight to hasten adhesiveness (Figure-1b). Next day normal culture medium was added to each flask. The normal culture medium contained DMEM supplemented with 10% FBS, penicillin (100 U/ml) and streptomycin (100 µg/ml). After 2 days, cells started to creep out from the bone pieces. The medium change was carried out twice a week. When 80-90% confluence was reached after 15 days, cells were transferred to new T-25 culture flask by using trypsinization methodology. Culture medium was removed from flask and cells were washed with 1× sterile PBS followed by addition of 2 ml of 0.25% trypsin-EDTA for 5 min. The enzymatic activity was stopped by adding 3 ml of culture medium, and the contents of flask were collected in a centrifuge tube and centrifuged at 2500 rpm for 5 min. The supernatant was discarded, pellet was suspended in culture medium and mixed gently to get single cell suspension and subcultured in a new T-25 flask at the rate of 5×10^3^-10^4^/cm^2^. The culture was maintained at 37°C, 5% CO_2_ and humidified atmosphere. After 7-8 days, cells reached in full confluence and further passage was done following trypsinization.

**Figure-2:**
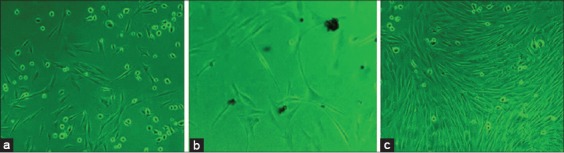
Observation under inverted phase contrast microscope. Fetal osteoblast cells showing trigonal, spindle to polygonal morphology after 10 days of culture (a) 10X; (b) 40X, (c) At passage 3, cells showing near 80% confluency (10X).

**Figure-3:**
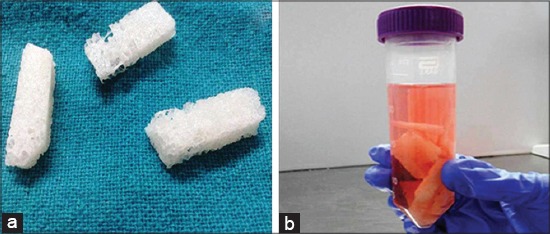
(a) Acellular cancellous bovine bone scaffold, (b) Incubation of scaffolds in DMEM (Pre-wetting) before seeding.

**Figure. 4:**
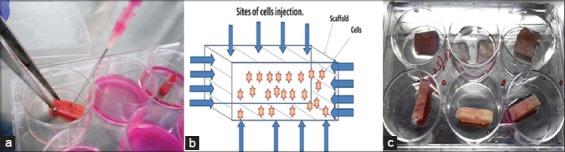
(a) Seeding of acellular scaffold, (b) Diagrammatic representation of seeding pattern, (c) Seeded scaffolds.

Scaffolds were incubated with culture medium for 4 h to pre-wet the scaffold surface. The second or third passaged fetal osteoblast cells were washed with sterile 1× PBS and detached with trypsinization methodology [2]. After centrifugation, cells pellet was suspended in culture medium and 4×10^6^ cells suspension in 100 µl culture medium were prepared and were seeded per scaffold. The cells were transferred to the core of scaffold with the help of 16 gauze needle and scaffolds were incubated after seeding for 4 h in incubator, and then culture media was added (Figure-4a, b). These scaffolds were cultured in 6-well plates for up to 2 weeks (Figure-4c). The cultures were maintained in a humidified atmosphere consisting 5% CO_2_ at 37°C, and the media was changed twice a week [22]. The scaffolds were routinely examined by an Olympus phase contrast microscope.

### Scanning electron microscope (SEM)

The osteoblast cell adhesion and penetration in porous matrix were assessed by SEM. The samples from tissue constructs that is fetal osteoblast seeded scaffold, acellular bone scaffold and native bovine bone were observed by SEM. The samples taken from the deep core of the cuboid scaffolds were washed in 1× PBS and fixed in 2.5% glutaraldehyde in PBS for overnight at 4°C. Then, they are dehydrated in a series of ethanol solutions (Merck, Germany). The samples were first incubated in 30, 50 and 70% ethanol for 10 min each, then in 90 and 100% ethanol for 15 min each. The tissue samples were then dried in a Critical Point Dryer using CO_2_ as the transitional fluid and mounted on SEM specimen holders. Specimens were mounted on aluminum stubs using adhesive silicon tape. After that, a thin layer of gold/palladium ion sputtering was done on Jeol ion sputter Model JFC 1600 at 7-10 mA and 1-2 KV for 15 min. Finally, the specimens were observed under SEM (JEOL, JSM 6610 LV, Japan) at appropriate acceleration voltage and the magnification range to test the adhesion, anchorage to pore, proliferation and matrix secretion of osteoblast cells on the acellular bovine bone scaffold.

### Histological analysis of tissue constructs

Osteoblast Seeded and acellular bone scaffolds were cut into small pieces and fixed in 10% formalin for 48-72 h. The scaffolds were decalcified in Goodling and Stewart’s fluid containing 15 ml formic acid, 5 ml formalin and 80 ml distilled water [23]. The completion of decalcification was assessed by flexibility, transparency and pin penetrability of the bone sections. The tissues were processed in a routine manner and 4 μm thick sections were cut and stained with hematoxylin and eosin (H and E) stain as per the standard procedure [24].

## Results

### Isolation, culture, and expansion of primary rabbit fetal osteoblasts

During primary culture of fetal osteoblast cells, after 2-3 days, round or polygonal cells were observed migrating from bone pieces under phase contrast microscope (Figure-1c). Initially, this culture represented mixture of heterologous cells population. Cells were observed having protuberances and adhering to culture flask after 5 days of culture. They exhibited morphologies ranging from triangular, polygonal to short spindle shapes (Figure-2a and b). After passage one, triangular cells were prevalent and packed closely in culture flask. Cell proliferation was uniform and throughout the culture flask (Figure-2c).

### Preparation of acellular cancellous bovine bone scaffold for seeding

After freeze and thaw cycles gross appearance of bone scaffolds changed, they became porous and white. After treatment with 3% H_2_O_2_ and 70% ethanol, scaffolds became more clear and sterilized (Figure-3a). Scaffolds were incubated with culture medium for 4 h to pre-wet the scaffold surface (Figure-3b).

### Seeding of osteoblasts on decellularized bovine cancellous bone scaffold

**Figure-5:**
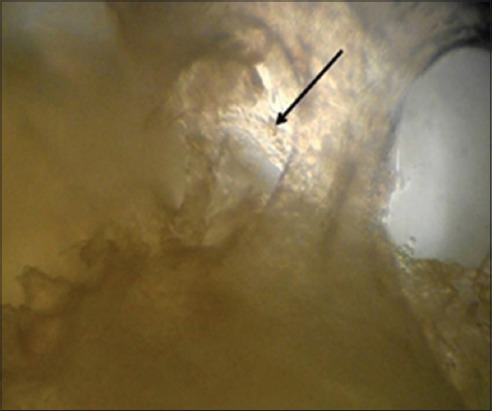
Seeded scaffold showing the matrix and cells after 10 days (10X) under inverted phase contrast microscope.

Acellular scaffold was then seeded on the scaffolds. The active seeding of osteoblasts on scaffolds by multiple injection techniques with a 16G hypodermic needle proved to be technically feasible and two to three days after seeding cells adhered to ECM. The cells proliferated and grew into the inner pores of the scaffold which were observed under phase contrast microscope (Figure-5). Grossly, color change of media in which scaffolds were cultured, was noticed after every 3-4 days, which was an indication of cell proliferation and viability. No contamination was observed in culture up to 14 days post-seeding.

### SEM

**Figure-6:**
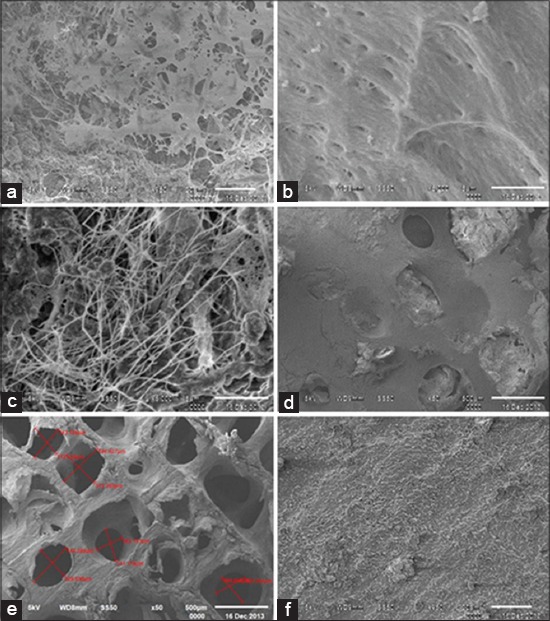
Showing SEM of (a) Seeded scaffold, (b) Acellular scaffold, (c) Native bone under higher magnification and (d) Seeded, (e) Acellular, (f) Native (under lower magnification).

The SEM was used to observe the architecture of matrix, pores and interconnectivity of pores of decellularized scaffold and osteoblasts adhesion and proliferation on matrix in case of seeded scaffold. In case of fresh cancellous bone under higher magnification 5000× and 2500×, we could see the arrangement of collagen fibers (Figure-6c). They appeared as a complex meshwork of wavy intercrossing collagen fibers. The collagen fibers were curled and irregular in arrangement. No pores were observed in the case of fresh cancellous bone under lower magnification (Figure-6f). Whereas, in case of acellular scaffold under lower magnification 250× to 50× many of pores were visible having pore diameter ranging between 200 and 500 µm (Figure-6e). Acellular bone appears as a highly porous scaffold under lower magnification, but in higher magnification, we could see collagen fiber which was less disorganized, closely and flatly packed (Figure-6b).

In case of seeded scaffold, SEM indicated that cells proliferated and grew in inner pores of the scaffold. Under higher magnification 5000×, cells adherence to the acellular scaffold was visible and cells adhered to scaffold material surface with processes and multiple filopodia (Figure-6a). Cells were found to spread and anchored well onto the pore wall. Under lower magnification, numerous pores filled with cellular material were visible (Figure-6d).

### Histological evaluation

**Figure-7:**
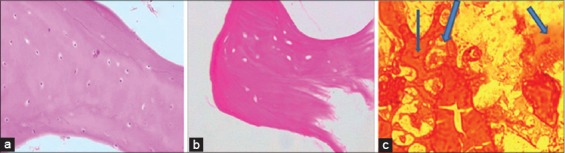
(a) Native bovine bone (H&E staining) (20X), (b) Acellular scaffold (H&E staining) (20X), (c) Seeded scaffold (Thick arrow is showing newly laid matrix) (H&E staining) (40X).

Histological examination of native, acellular and seeded graft was done after cutting section and H and E staining under routine procedure. In the case of native bovine bone, matrix had taken pink stain, and nuclei of osteocytes were clearly visible as blue dots within osteoid matrix. Stained section of freeze and thaw group showed nearly eliminated all cell nuclei. These cells were trapped inside lacunae in matrix (Figure-7a). In, acellular matrix we could have observe remaining intact porous matrix of concentric layer of collagen fibers showing removal of cells nuclei (Figure-7b). The matrix fibers were distorted. Histological section of seeded scaffold reveals that pores that were visible, occupied by newly formed osteoid that was appeared as lightly stained matrix, whereas decellularized matrix stained dark pink (Figure-7c).

## Discussion

During primary culture of fetal rabbit osteoblast, round and some polygonal cells were found to migrate from bone pieces. They started to attach on culture flask surface, and their morphology became triangular to short spindle shape [25]. Bone ECM retains the natural three-dimensional space for proliferation, differentiation, and osteogenesis of the cells. The biocompatibility of scaffold directly affects the adhesion, growth, and proliferation of the cell. A study has shown that natural bone ECM derived from pig was conducive to cell adhesion, growth and provided good space and surface for the secretion of ECM, and rabbit osteoblast cells could be attached and grew well [26]. In the present study, a better and complete processing of the scaffold led to proper cell adhesion and proliferation. Evidence of human osteoblast cell viability, proliferation, and osteocalcin synthesis on solvent dehydrated cancellous bone matrix is also present [2]. It is important to investigate the relationship between pore parameters and cell seeding. The previous studies have shown that pore sizes and porosities have an effect on cell proliferation [27]. It was found that the pores with a size of 300-400 µm were the best for cell attachment and showed more cells in the scaffolds, and the larger pores resulted in a decrease of the cell numbers in the scaffolds and indicates that if the pores are larger than 400 µm, some of the cells cannot be caught in the scaffolds during the seeding, so 300-400 µm might be the appropriate pore size for cell seeding. Acellular scaffolds used for seeding in our study have achieved pores size 200-500 µm that has been revealed in SEM results. Other studies have shown well proliferation and culture of osteoblasts [2] for 21 days, for 14 days [15], and bone marrow-derived human stem cells cultured for 5 weeks on bovine acellular matrixes [28]. Studies have indicated that maturation of grafts *in vitro* cultivation enhances bone healing after implantation [29], as compared to implantation of the acellular scaffold and scaffolds seeded with cells immediately before surgery. Bovine scaffold was found to be biocompatible for cells attachment as the matrix-supported osteoblast survival and proliferation over a period of 14-day in *in vitro* culture media, found in SEM results in the present study. Scanning electron micrographs of tissue constructs have also shown proliferation of osteoblast on acellular porous matrix. The viability of the cells can be ascertained by observing a typical cell matrix with filopodial extensions of the cells. Filopodia and cell processes forming a cell sheet over matrix were evident. Their growth and attachment have well shown in several studies [2,15,22]. Acellular scaffold appears as highly porous material having pores and on higher magnification when surface collagen was compared with fresh bovine matrix, reveal closely and densely packed collagen matrix. Collagen fibers were less disoriented and densely arranged in comparison to fresh bone sample [30].

H and E staining revealed porous matrix in case of acellular matrix where no cells and nuclei were observed when compared to section of fresh cancellous bone in which numerous cells containing nuclei were visible. As in one study, bovine cancellous bone decellularized by some anionic detergent revealed this vanished cellular portion from matrix on H and E staining [31]. Whereas, in the case of seeded scaffold we could see newly laid down matrix inside the pores of acellular matrix as reported in other studies. H and E staining revealed new bone formation inside matrix of osteogenic induced sheep marrow cells seeded hydroxyapatite scaffold and rat osteoblast seeded on bovine matrices [15,32].

## Conclusion

This study provides an evidence of cell viability and adhesion on acellular bovine cancellous matrix. Our *in vitro* results suggest acellular bovine matrix may be a suitable template for proliferation of osteoblast and for making a composite bone graft substitute. The multiple injection techniques for cell seeding in a definite pattern could successfully and uniformly seed the scaffold up to core of the tissue in this study. Hence, these cell-biomaterial tissue construct may provide a better option in bone grafting procedure.

## Authors’ Contributions

Rashmi: Carried out the research work. RP: Contributed to planning and designing this work. Amarpal, HPA, PK and AMP: Helped in interpretation and to correct the manuscript. AKT: Helped in microscopic evaluation. PS, PT and ABM: Helped in graft making process. All authors read and approved the final manuscript.
